# Supplementary material for: Ser14 phosphorylation of Bcl-xL mediates compensatory cardiac hypertrophy in male mice
Source: Nat Commun. 2023 Sep 19;14:5805. doi: 10.1038/s41467-023-41595-x (PMC10509265; doi:10.1038/s41467-023-41595-x)

Supplementary Information

Ser14 phosphorylation of Bcl-xL mediates compensatory cardiac hypertrophy in male mice

Michinari Nakamura[1*], Mariko Aoyagi Keller[1], Nadezhda Fefelova[1], Peiyong Zhai[1], Tong Liu[2], Yimin Tian[1], Shohei Ikeda[1], Dominic P Del Re[1], Hong Li[2], Lai-Hua Xie[1], Junichi Sadoshima[1*]

Affiliation:

1. Department of Cell Biology and Molecular Medicine, Cardiovascular Research Institute, Rutgers-New Jersey Medical School, 185 South Orange Ave, Newark, NJ 07103
2. Center for Advanced Proteomics Research, Department of Biochemistry & Molecular Biology, Rutgers New Jersey Medical School, Newark, NJ, 07103

*Correspondence to

Department of Cell Biology and Molecular Medicine, Cardiovascular Research Institute,

Rutgers-New Jersey Medical School, 185 South Orange Ave, MSB G-609, Newark, NJ 07103

+1-973-972-8619

E-mail: nakamumi@njms.rutgers.edu (M.N.), sadoshju@njms.rutgers.edu (J.S.)

Supplementary Table 1

	Biocarta pathways	SIZE	NES	NOM p-val	FDR q-val
1	BIOCARTA_**FCER1_PATHWAY**	37	1.75	0	0.322
2	BIOCARTA_**ERK_PATHWAY**	25	1.75	0	0.184
3	BIOCARTA_IL1R_PATHWAY	26	1.74	0.087	0.138
4	BIOCARTA_**INTEGRIN_PATHWAY**	31	1.7	0	0.126
5	BIOCARTA_VIP_PATHWAY	23	1.66	0	0.138
6	BIOCARTA_BCR_PATHWAY	31	1.64	0	0.144
7	BIOCARTA_TFF_PATHWAY	19	1.63	0	0.13
8	BIOCARTA_TCR_PATHWAY	37	1.62	0	0.126
9	BIOCARTA_BARRESTIN_SRC_PATHWAY	16	1.6	0	0.149
10	BIOCARTA_FMLP_PATHWAY	30	1.6	0	0.139
11	BIOCARTA_HDAC_PATHWAY	22	1.59	0	0.136
12	BIOCARTA_MET_PATHWAY	31	1.59	0	0.132
13	BIOCARTA_IL2RB_PATHWAY	32	1.59	0	0.125
14	BIOCARTA_BAD_PATHWAY	22	1.58	0	0.12
15	BIOCARTA_HIVNEF_PATHWAY	52	1.57	0	0.115
16	BIOCARTA_NFKB_PATHWAY	19	1.56	0.094	0.114
17	BIOCARTA_GPCR_PATHWAY	28	1.53	0	0.125
18	BIOCARTA_RAC1_PATHWAY	20	1.5	0	0.132
19	BIOCARTA_PDGF_PATHWAY	28	1.49	0	0.131
20	BIOCARTA_EIF_PATHWAY	16	1.49	0	0.13

Supplementary Table 1. Gene sets enriched in the heart after TAC compared to after sham operation in WT mice. ERK1/2 were identified as significantly activated kinases in the heart during acute pressure overload. Among the top 4 gene sets enriched in TAC vs. Sham, ERK1/2 are involved in 3 Biocarta pathways, including the FCER1 pathway, ERK pathway, and Integrin pathway (as shown in bold). NES, normalized enrichment score. GSEA nominal *p* value is the statistical significance of the enrichment score by using a phenotype-based permutation test with no adjustment. FDR (false discovery rate)-adjusted q value for each pathway is indicated.

Supplementary Figure 1

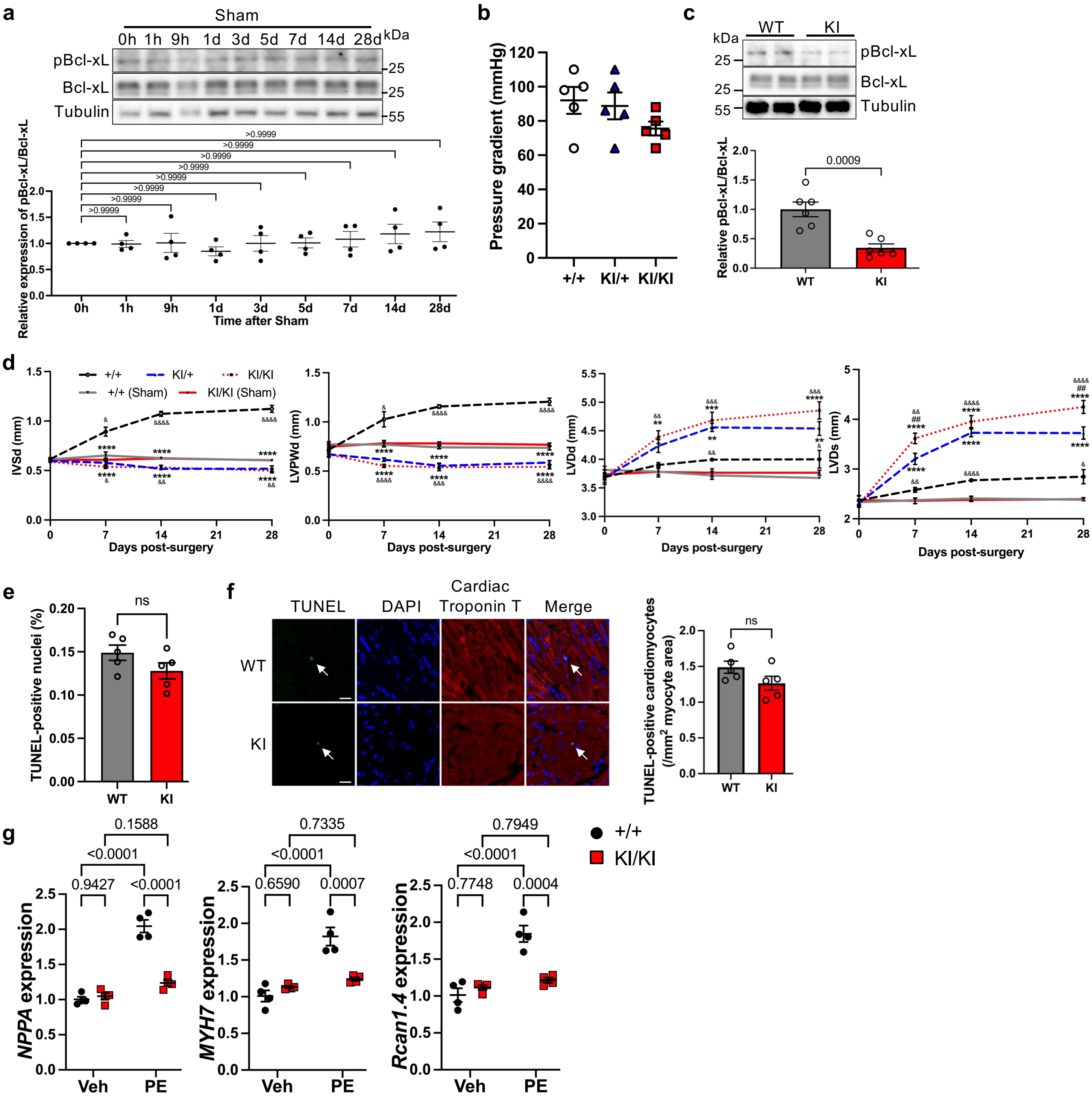

Supplementary Figure 1 Bcl-xL knock-in (KI) mice in which Serine (Ser) 14 of Bcl-xL is replaced with Alanine, develop more severe cardiac dysfunction in response to pressure overload than control wild type (WT) mice. (**a**) Representative immunoblots showing phosphorylation of Bcl-xL at Ser14 in the heart with time course after Sham. h; hours, and d; days after Sham surgery. Lower panel shows densitometric analysis of relative expression of pBcl-xL (Ser14)/Bcl-xL in the heart. Kruskal-Wallis test with sham 0 h as the control. *p* values are shown in the figure (n=4). (**b**) Pressure gradient between femoral artery and ascending aorta. One-way ANOVA (n=5). (**c**) Immunoblots showing phosphorylation of Bcl-xL at Ser14 and tubulin as a loading control in the WT and Bcl-xL (S14A) homozyogous KI mouse heart one hour after TAC. Lower panel shows densitometric analysis (n=6). Two-sided unpaired *t* test. (**d**) Echocardiographic parameters of interventricular septum at end-diastole (IVSd), left ventricular (LV) posterior wall at end-diastole (LVPWd), and LV diameter at end-diastole (LVDd) and end-systole (LVDs) (n=6-16 for TAC and 5 for Sham). Two-way ANOVA with Tukey's multiple comparison test. *n* represents biologically independent replicates. **** $p < 0.0001$ and ** $p < 0.01$ compared to +/+ mice. ## $p < 0.01$ compared to KI/+ mice. &&&& $p < 0.0001$, &&& $p < 0.001$, && $p < 0.01$ and & $p < 0.05$ compared to Sham (two-sided upaired *t* test or Mann-Whitney test). (**e**) The percentage of TUNEL positive nuclei in the hearts of S14A KI and WT mice four weeks post-TAC. n=5. Two-sided unpaired *t* test. ns not significant. (**f**) Co-staining of WT and S14A KI mouse hearts 2 weeks after TAC with terminal deoxynucleotidyl transferase dUTP nick end labeling (TUNEL) and cardiac troponin T. Arrows indicate TUNEL-positive cardiomyocytes. Scale bar; 20 **μ**m. Right panel shows quantitative analysis of TUNEL-positive cardiomyocytes. n=5. Two-sided unpaired *t* test. ns not significant. (**g**) Relative *NPPA*, *MYH7*, and *Rcan1.4* gene expressions in response to 100 **μ**M phenylephrine (PE) or vehicle for 24 h in adult cardiomyocytes isolated from WT and homozygous S14A KI mice (n=4). Two-way ANOVA with Tukey's multiple comparison test. n represents biologically independent replicates. Adjusted *p* values (d): 0.0098 (LVDd, 7 days, +/+ vs KI/KI), 0.0011 (LVDd, 14 days, +/+ vs KI/+), 0.0005 (LVDd, 14 days, +/+ vs KI/KI), 0.0055 (LVDd, 28 days, +/+ vs KI/+), 0.0074 (LVDs, 7 days, KI/+ vs KI/KI), 0.0032 (LVDs, 28 days, KI/+ vs KI/KI). *p* values for (d): 0.0177 (IVSd, 7 days, +/+, Sham vs TAC), 0.0159 (IVSd, 7 days, KI/KI, Sham vs TAC), 0.0057 (IVSd, 14 days, KI/KI, Sham vs TAC), 0.0019 (IVSd, 28 days, KI/KI, Sham vs TAC), 0.0216 (LVPWd, 7 days, +/+, Sham vs TAC), 0.0002 (LVPWd, 14 days, KI/KI, Sham vs TAC), 0.0028 (LVDd, 14

days, +/+, Sham vs TAC), 0.0303 (LVDd, 28 days, +/+, Sham vs TAC), 0.0026 (LVDd, 7 days, KI/KI, Sham vs TAC), 0.0005 (LVDd, 14 days, KI/KI, Sham vs TAC), 0.0001 (LVDd, 28 days, KI/KI, Sham vs TAC), 0.0061 (LVDs, 7 days, +/+, Sham vs TAC), 0.018 (LVDs, 28 days, +/+, Sham vs TAC), 0.0025 (LVDs, 7 days, KI/KI, Sham vs TAC). Data are mean ± SEM. Source data are provided as a Source Data file.

Supplementary Figure 2

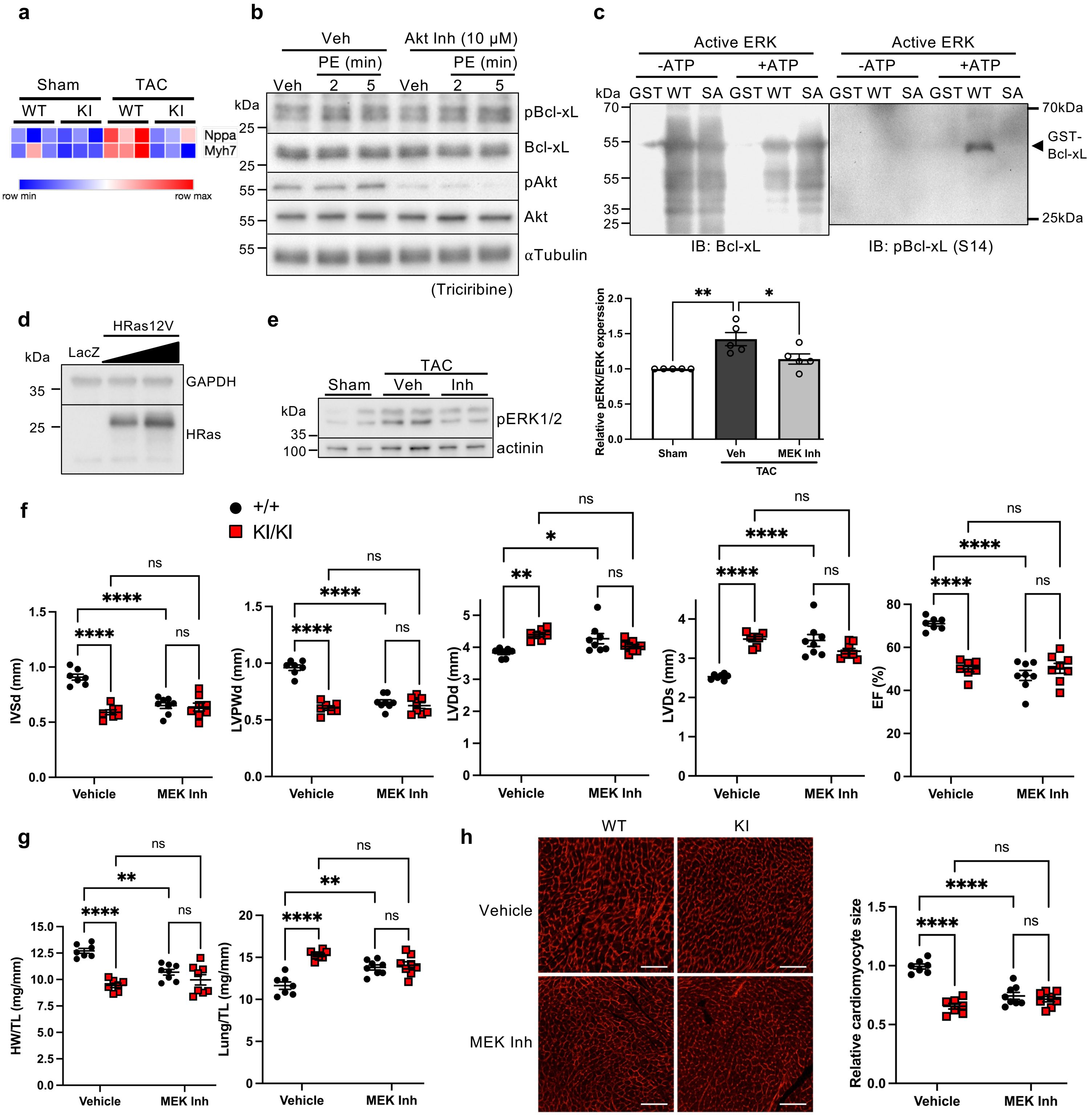

Supplementary Figure 2 Inhibition of the MEK-ERK pathway suppresses hypertrophy and decreases systolic function in wild type (WT) but not Bcl-xL-S14A homozyogous knock-in (KI) mice. (**a**) Heatmap of *NPPA* and *Myh7* gene expressions in WT and KI mouse hearts using the RNA-sequencing data (related to Fig. 3a). (**b**) Representative immunoblots showing pBcl-xL (Ser14) in cardiomyocytes treated with an Akt inhibitor (Triciribine) or vehicle following treatment with phenylephrine (PE) for the indicated times (minutes). Repeated three times. (**c**) *In vitro* kinase assay using active ERK1 and recombinant Bcl-xL-WT, S14A mutant, or GST alone, followed by blots with Bcl-xL and Bcl-xL Ser14 phospho-specific antibody. Repeated twice. (**d**) Immunoblots showing the expression of H-Ras and GAPDH as a loading control in adult cardiomyocytes transduced with adenovirus harboring H-Ras or LacZ. Repeated twice. (**e**) Representative immunoblots showing pERK1/2 and its quantification in WT mouse hearts after 9 hours of TAC. PD0325901 was used to inhibit MEK. One-way ANOVA followed by Tukey's multiple comparison test (n=5). Adjusted *p* values: 0.0023 (Sham vs TAC-Vehicle), 0.0309 (TAC-Vehicle vs TAC-MEK Inh). (**f**) Echocardiographic parameters of the interventricular septum at end-diastole (IVSd), left ventricular (LV) posterior wall at end-diastole (LVPWd), LV diameter at end-diastole (LVDd) and end-systole (LVDs), and ejection fraction (EF) 1 week after TAC. n=7 (Vehicle) and 8 (MEK Inhibitor). Adjusted *p* values: 0.003 (LVDd, Vehicle, +/+ vs KI/KI), 0.0213 (LVDd, +/+, Vehicle vs MEK Inh). (**g**) Heart weight (HW) normalized by tibia length (TL) and lung weight normalized by TL 1 week after TAC. n=7 (Vehicle) and 8 (MEK Inhibitor). Adjusted *p* values: 0.0011 (HW/TL, +/+, Vehicle vs MEK Inh), 0.0013 (Lung/TL, +/+, Vehicle vs MEK Inh). (**h**) Wheat Germ Agglutinin (WGA) staining of the indicated heart tissues and quantification analysis of relative cardiomyocyte size. Scale bar; 100 μm. n=7 (Vehicle) and 8 (MEK Inhibitor). Two-way ANOVA with Tukey's multiple comparison test unless otherwise

stated. In all graphs, **** $p < 0.0001$, ** $p < 0.01$, * $p < 0.05$, ns not significant. n represents biologically independent replicates unless otherwise indicated. Data are mean ± SEM. Source data are provided as a Source Data file.

Supplementary Figure 3

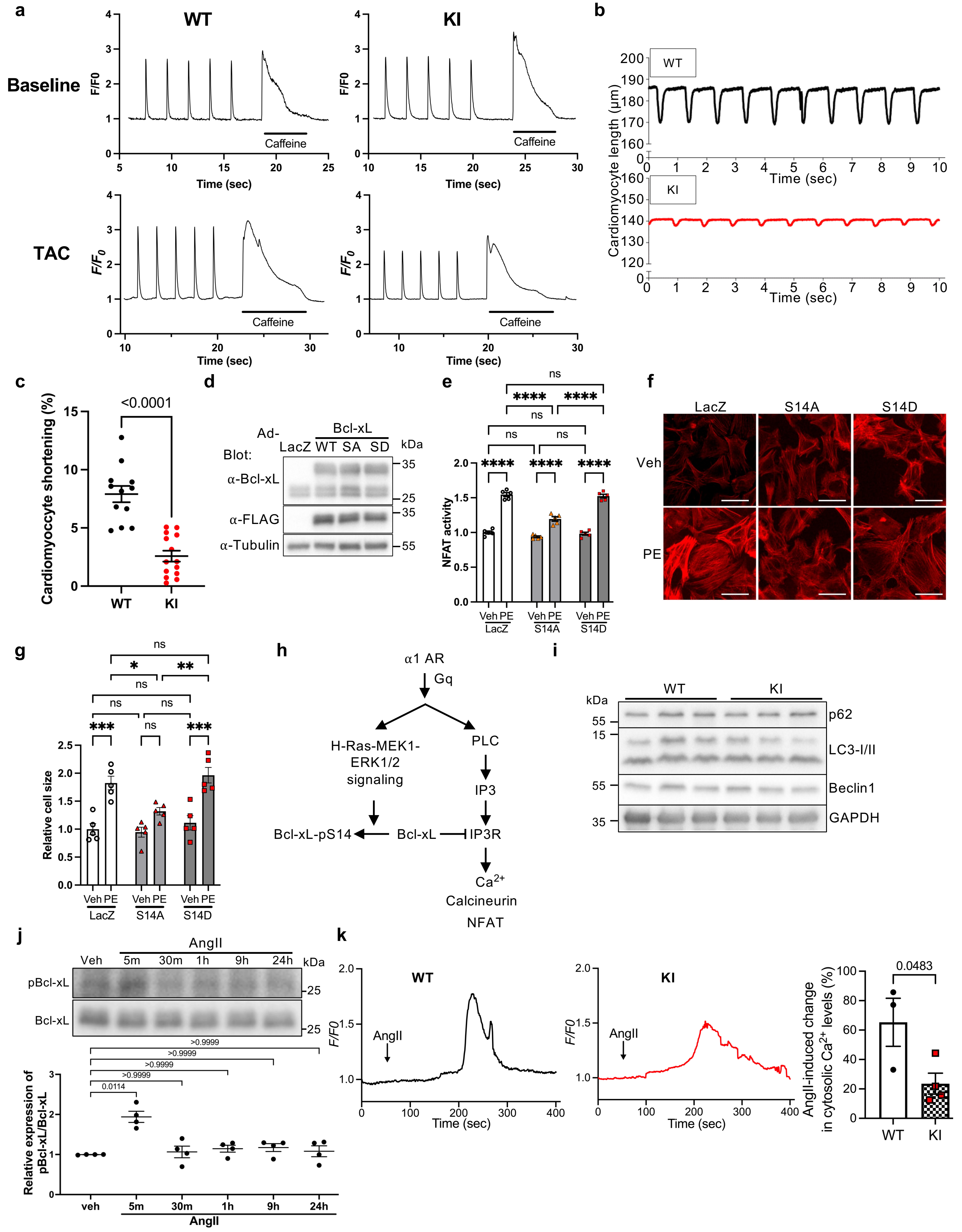

Supplementary Figure 3 Wild type (WT) but not Bcl-xL-S14A homozygous knock-in (KI) cardiomyocytes exhibit increased Ca^{2+} transient amplitude and sarcoplasmic reticulum (SR) Ca^{2+} content after TAC-induced pressure overload. (a) Representative traces of Ca^{2+} transient (*F/F0*) in WT and KI cardiomyocytes after1 day of TAC or at baseline. (**b**) Representative traces of sarcomere shortening in cardiomyocytes isolated from WT and KI mouse hearts after two days of TAC. (**c**) Quantification analysis of sarcomere shortening (5-7 cells/heart from 2 hearts/group, WT n=12, KI n=14). Two-sided unpaired *t* test. (**d**) Immunoblots showing the expression of FLAG-Bcl-xL-WT and -S14A and -S14D mutants in cardiomyocytes. Repeated three times. (**e**) Relative NFAT activity in cardiomyocytes expressing Bcl-xL-S14A, -S14D, or LacZ in response to phenylephrine (PE). (n=5 independently prepared cardiomyocyte preparations/cultures). (**f**) Phalloidin staining for determination of size of rat neonatal cardiomyocytes expressing Bcl-xL-S14A, -S14D, or LacZ in the presence or absence of PE. Scale bar; 50 µm. (**g**) Quantification analysis of cardiomyocyte size (n=5 independently prepared cardiomyocyte preparations/cultures). Adjusted *p* values: 0.0367 (PE, LacZ vs S14A), 0.0045 (PE, S14A vs S14D), 0.0003 (LacZ, Veh vs PE), 0.0002 (S14D, Veh vs PE). (**h**) Our proposed signaling pathway, involving Bcl-xL phosphorylation in hypertrophic stimuli-induced calcium-calcineurin-NFAT signaling. Phosphorylation of Bcl-xL at Ser14 by ERK disrupts its inhibitory interaction with IP3R, thereby augmenting Ca^{2+} signaling to promote compensatory hypertrophy in response to pressure overload. (**i**) Immunoblots showing the levels of autophagy-related proteins in the hearts of S14A homozygous KI and WT mice at baseline. (**j**) Representative immunoblots showing phosphorylation of Bcl-xL at Ser14 in WT mouse neonatal cardiac fibroblasts with time course after 50 nM angiotensin II (AngII) stimulation. m; minutes and h; hours. Lower panel shows densitometric analysis of relative expression of pBcl-xL

(Ser14)/Bcl-xL. Kruskal-Wallis test with vehicle as the control. p values are shown in the figure (n=4, independently prepared cells). (**k**) Representative traces of cytosolic Ca^{2+} levels (*F/F0*) in WT and S14A homozygous KI mouse cardiac fibroblasts with AngII stimulation. Right panel shows quantitative analysis of AngII-induced changes in cytosolic Ca^{2+} levels (n=3-4, independently prepared cells). Two-sided unpaired t test. Two-way ANOVA with Tukey's multiple comparison test unless otherwise stated. **** $p < 0.0001$, *** $p < 0.001$, ** $p < 0.01$, * $p < 0.05$, ns not significant. Data are mean ± SEM. Source data are provided as a Source Data file.

Supplementary Figure 4

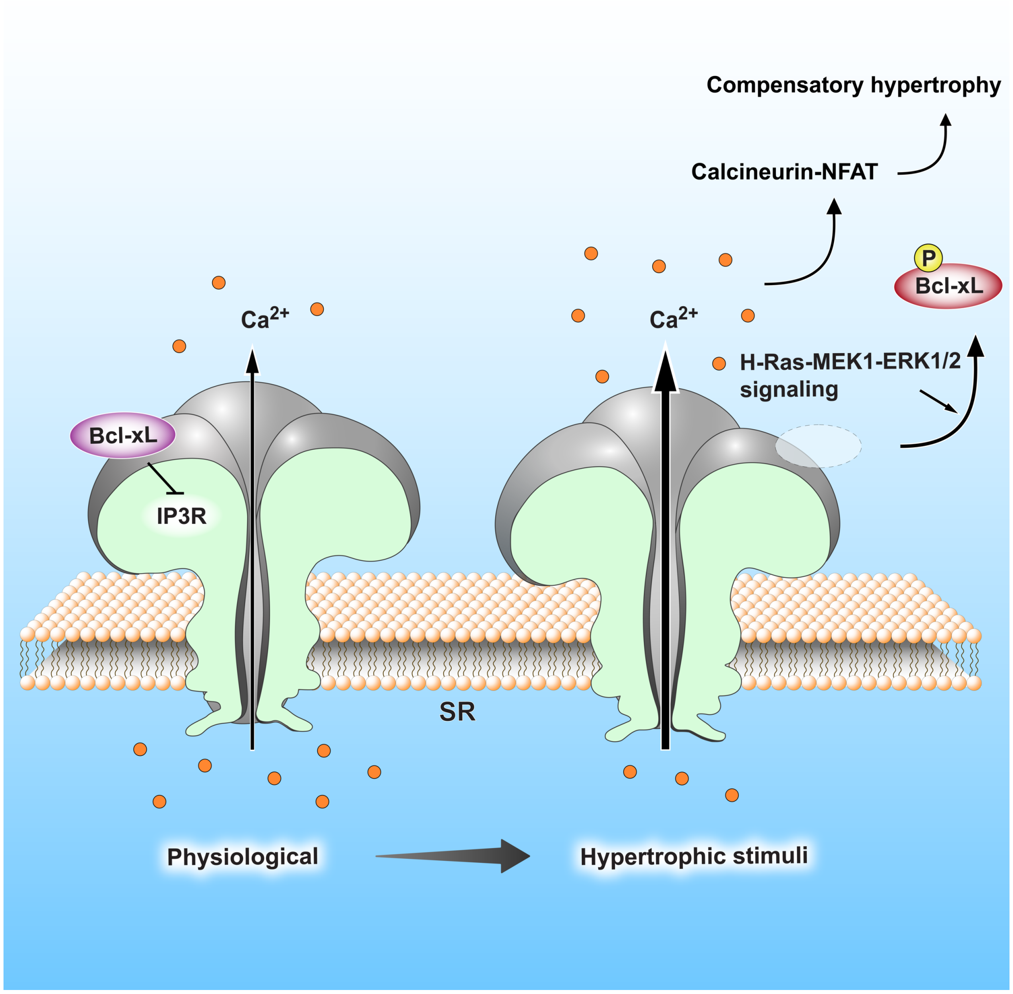

Supplementary Figure 4 Schema of our proposed mechanism. Activation of the MEK1-ERK1/2 pathway by hypertrophic stimuli promotes Bcl-xL-Ser14 phosphorylation, which disrupts its inhibitory interaction with IP3R, thereby augmenting Ca^{2+} release from the sarcoplasmic reticulum (SR) and calcineurin-NFAT signaling. This mechanism is crucial for the development of adaptive hypertrophy to suppress wall stress and prevent acute decompensated heart failure.

Supplementary Figure 5 Uncropped Western blot images

Supplementary Figure 5 Uncropped scan images of immunoblots membrane

Fig. 1a

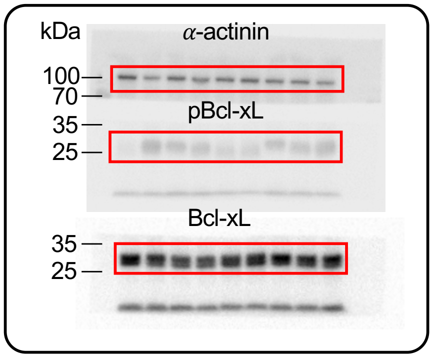

Fig. 2h

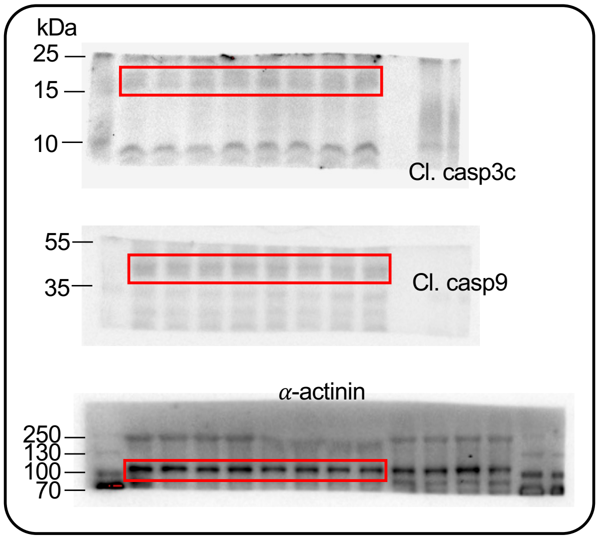

Fig. 3f

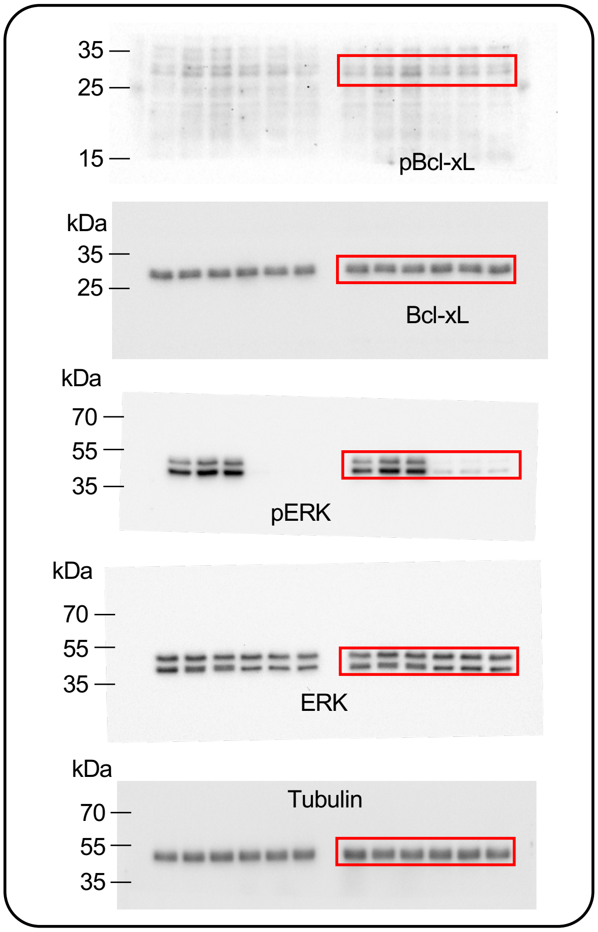

Fig. 3c

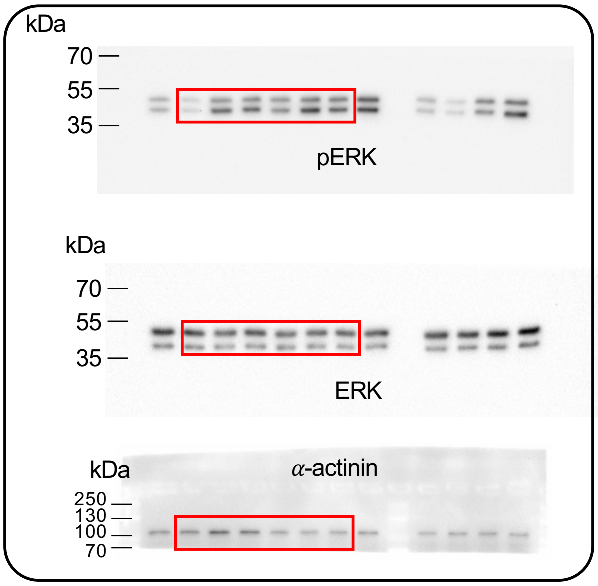

Fig. 3d

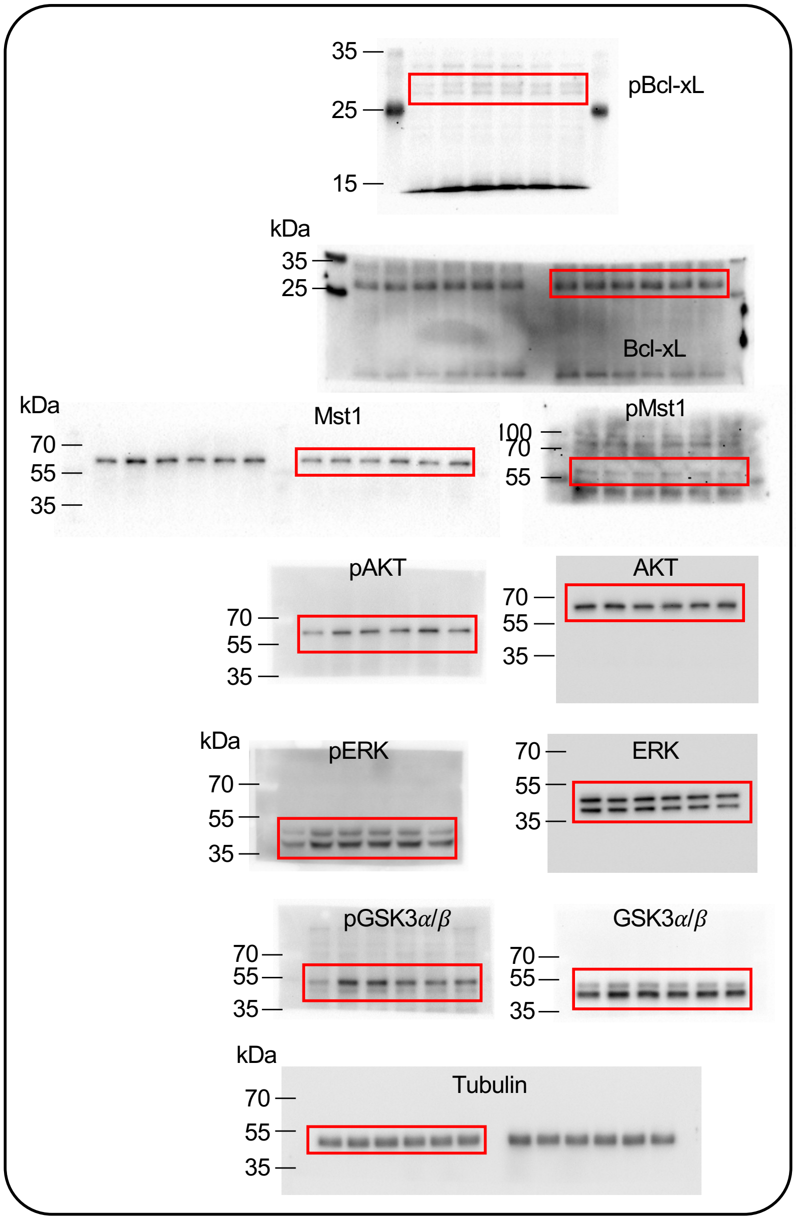

Fig. 3i

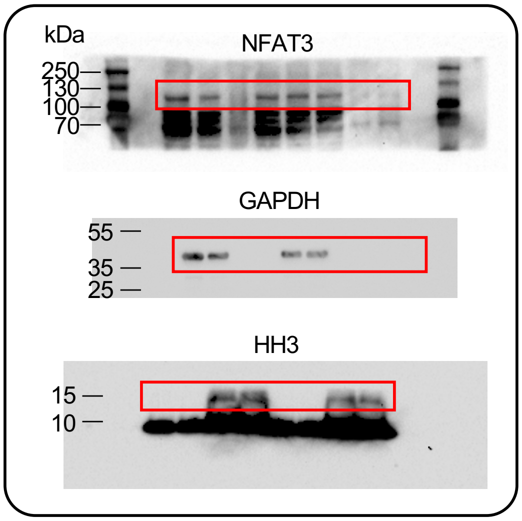

Fig. 3k

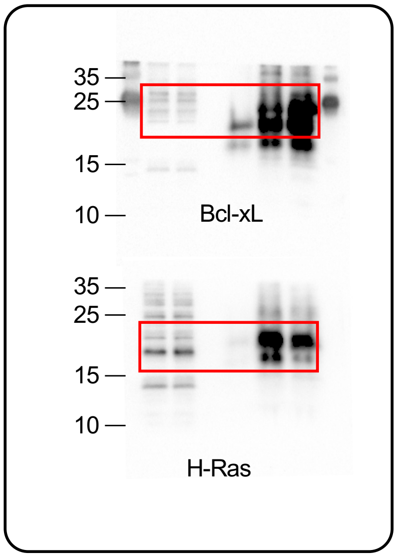

Fig. 3l

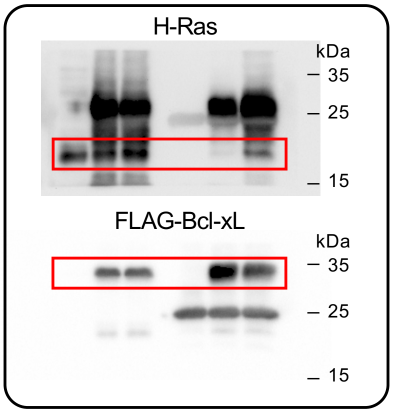

Fig. 5c

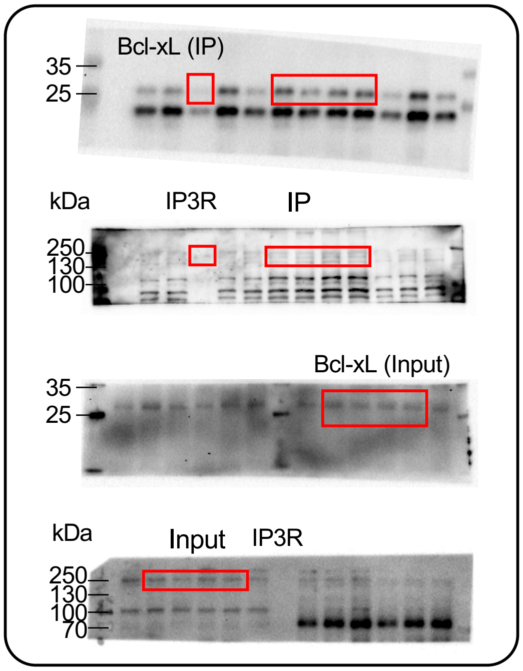

Fig. 5d

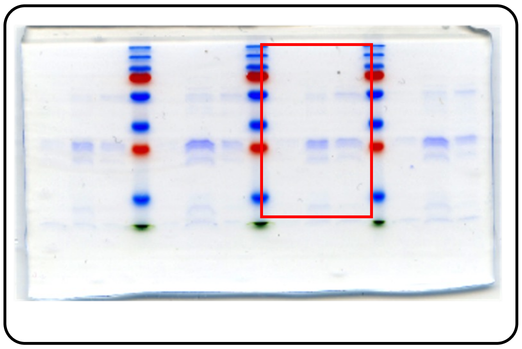

Supplementary Fig. 1a

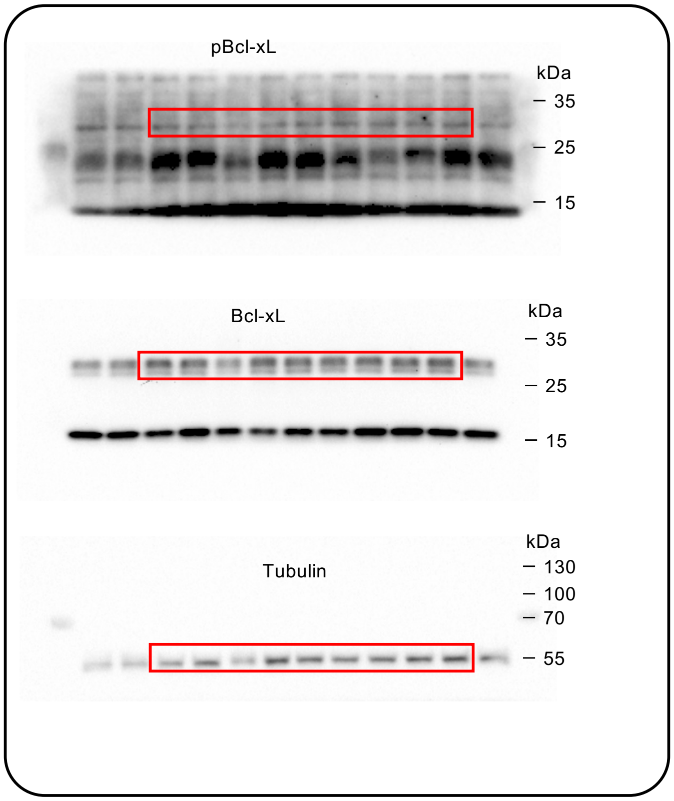

Supplementary Fig. 1c

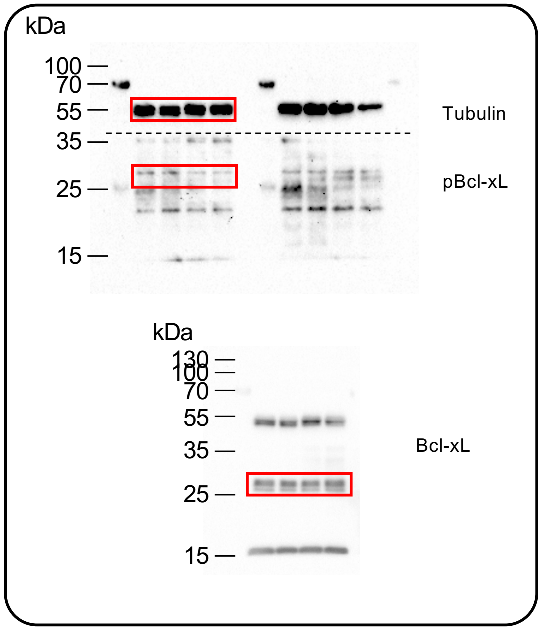

Supplementary Fig. 2b

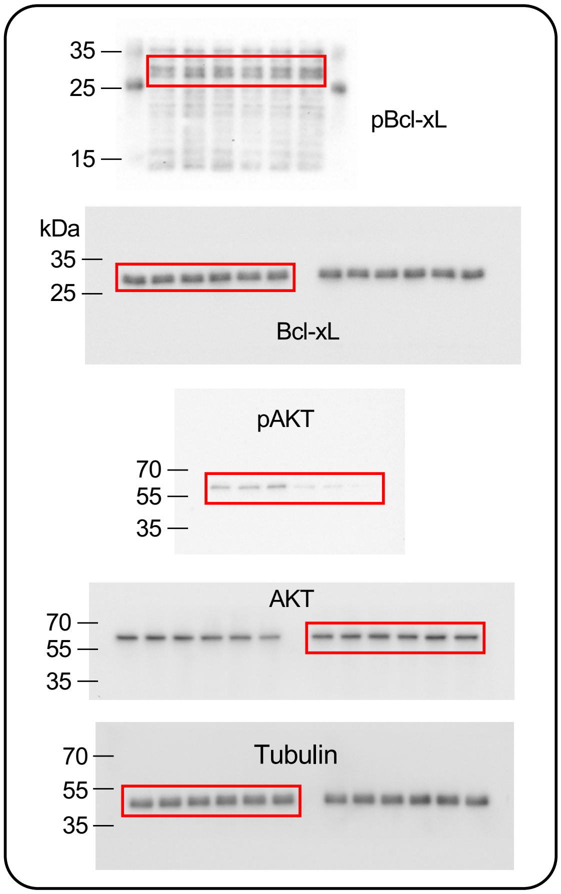

Supplementary Fig. 2c

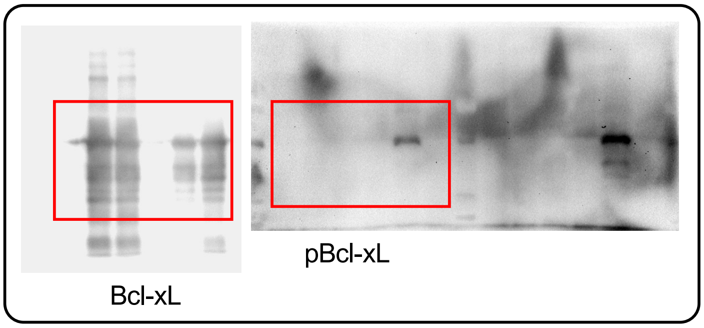

Supplementary Fig. 2d

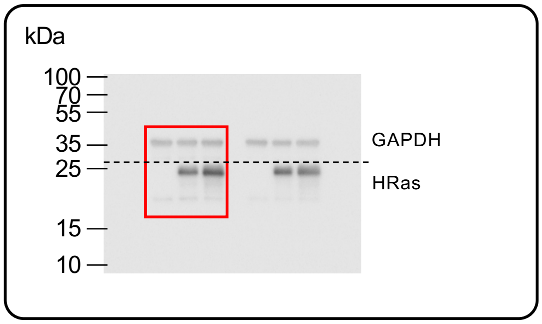

Supplementary Fig. 2e

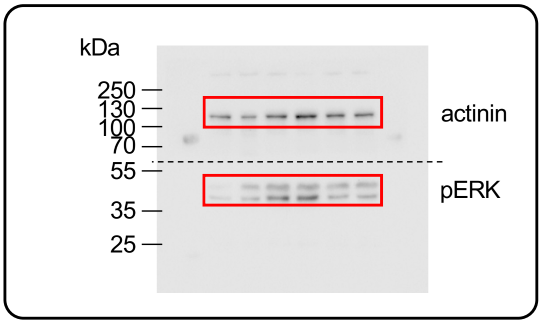

Supplementary Fig. 3d

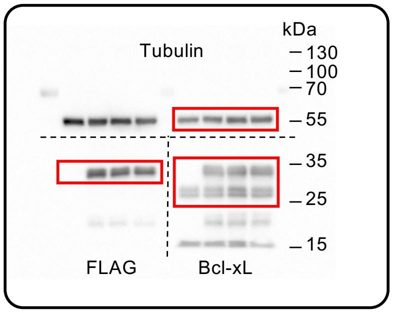

Supplementary Fig. 3i

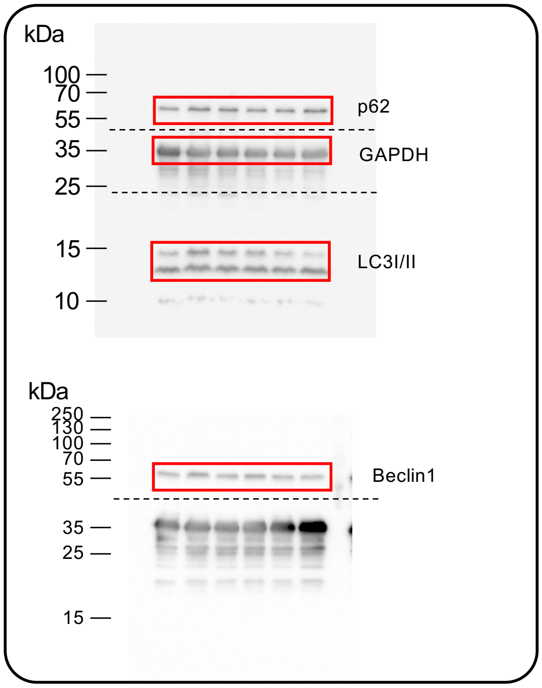

Supplementary Fig. 3j

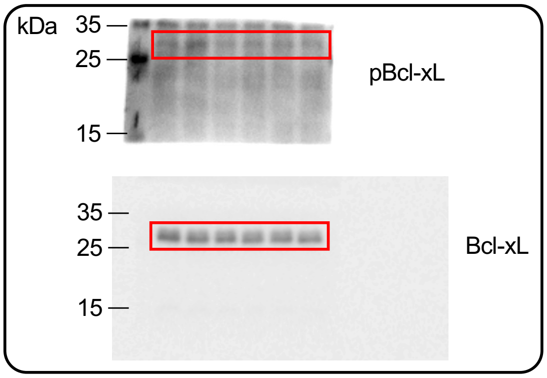

Supplement: Supplementary file 1 — Supplementary Information [file 41467_2023_41595_MOESM1_ESM.pdf]
