# Supplementary material for: Ser14 phosphorylation of Bcl-xL mediates compensatory cardiac hypertrophy in male mice
Source: Nat Commun. 2023 Sep 19;14:5805. doi: 10.1038/s41467-023-41595-x (PMC10509265; doi:10.1038/s41467-023-41595-x)
Supplement: Supplementary file 3 — Reporting Summary [file 41467_2023_41595_MOESM3_ESM.pdf]

nature portfolio

Corresponding author(s): Michinari Nakamura, Junichi Sadoshima

Last updated by author(s): Aug 31, 2023

Reporting Summary

Nature Portfolio wishes to improve the reproducibility of the work that we publish. This form provides structure for consistency and transparency in reporting. For further information on Nature Portfolio policies, see our Editorial Policies and the Editorial Policy Checklist.

Statistics

For all statistical analyses, confirm that the following items are present in the figure legend, table legend, main text, or Methods section.

n/a	Confirmed	
☐	☒	The exact sample size (*n*) for each experimental group/condition, given as a discrete number and unit of measurement
☐	☒	A statement on whether measurements were taken from distinct samples or whether the same sample was measured repeatedly
☐	☒	The statistical test(s) used AND whether they are one- or two-sided<br>*Only common tests should be described solely by name; describe more complex techniques in the Methods section.*
☒	☐	A description of all covariates tested
☐	☒	A description of any assumptions or corrections, such as tests of normality and adjustment for multiple comparisons
☐	☒	A full description of the statistical parameters including central tendency (e.g. means) or other basic estimates (e.g. regression coefficient) AND variation (e.g. standard deviation) or associated estimates of uncertainty (e.g. confidence intervals)
☐	☒	For null hypothesis testing, the test statistic (e.g. *F*, *t*, *r*) with confidence intervals, effect sizes, degrees of freedom and *P* value noted<br>*Give P values as exact values whenever suitable.*
☒	☐	For Bayesian analysis, information on the choice of priors and Markov chain Monte Carlo settings
☒	☐	For hierarchical and complex designs, identification of the appropriate level for tests and full reporting of outcomes
☒	☐	Estimates of effect sizes (e.g. Cohen's *d*, Pearson's *r*), indicating how they were calculated

Our web collection on statistics for biologists contains articles on many of the points above.

Software and code

Policy information about availability of computer code

Data collection	Data collection method is described in the Method section.<br>Software used:<br>Imaging Workbench v6 software<br>pCLAMP 10 Electrophysiology Data Acquisition and Analysis software<br>Nikon NIS-Elements imaging software
Data analysis	Data analysis method is described in the Method section.<br>Software used:<br>GraphPad Prism 9 Software<br>HISAT2 v2.2.1<br>HTSeq v0.6.1<br>edgeR v3.18.1<br>limma v3.32.10<br>R version v4.1.1<br>GSEA v4.1.0<br>Proteome Discoverer platform v2.4<br>ImageJ software (NIH) (https://imagej.nih.gov/ij/download.html)<br>Imaging Workbench v6 software<br>pCLAMP 10 Electrophysiology Data Acquisition and Analysis software

For manuscripts utilizing custom algorithms or software that are central to the research but not yet described in published literature, software must be made available to editors and reviewers. We strongly encourage code deposition in a community repository (e.g. GitHub). See the Nature Portfolio guidelines for submitting code & software for further information.

Data

Policy information about availability of data

All manuscripts must include a data availability statement. This statement should provide the following information, where applicable:

- Accession codes, unique identifiers, or web links for publicly available datasets
- A description of any restrictions on data availability
- For clinical datasets or third party data, please ensure that the statement adheres to our policy

The source data generated in this study is available as the Source Data file attached with the manuscript. RNA-sequencing data have been deposited at gene expression omnibus (GEO) and are publicly available as of the date of publication with accession numbers: GSE199705. The mass spectrometry data have been deposited to the ProteomeXchange Consortium via the PRIDE partner repository with the accession code PXD045118.

Human research participants

Policy information about studies involving human research participants and Sex and Gender in Research.

Reporting on sex and gender	N/A
Population characteristics	N/A
Recruitment	N/A
Ethics oversight	N/A

Note that full information on the approval of the study protocol must also be provided in the manuscript.

Field-specific reporting

Please select the one below that is the best fit for your research. If you are not sure, read the appropriate sections before making your selection.

☒ Life sciences ☐ Behavioural & social sciences ☐ Ecological, evolutionary & environmental sciences

For a reference copy of the document with all sections, see nature.com/documents/nr-reporting-summary-flat.pdf

Life sciences study design

All studies must disclose on these points even when the disclosure is negative.

Sample size	The sample size used in this study reflects the minimum number needed to achieve statistical significance. The sample size required was estimated to be n = 5-8 per group according to the power analysis based upon our previous studies examining the effects of pressure overload on cardiac hypertrophy and hypertrophic signaling. These studies include, but are not limited to, reference 5 (Ikeda, S. et al. Circ Res 2019), reference 13 (Nakamura, M et al. Cardiovasc Res 2021), reference 31 (Ikeda, S. et al. JACC Basic Transl Sci 2019), and reference 38 (Matsuda, T. et al. Circ Heart Fail 2017) of this paper.
Data exclusions	Mice that did not survive during the procedure of TAC surgery were excluded, which applies to the TAC-associated data collection in Figure 1 and 2 and Supplementary Figure 1 and 2.
Replication	In vivo experimental findings were reproducible as shown across multiple animals with a minimal of 2 surgical cohorts. In vitro experimental findings were independently reproduced at least 3 times unless specified. All attempts at replication were successful.
Randomization	Mice were not randomized because they were genetically identical within groups. The relevant experimental controls were used in each experiment as described in this paper. For experiments other than those involving mice, all samples, such as cells, were randomly allocated into groups and treated in the same manner across conditions.
Blinding	TAC surgeries and data collection and analysis for in vivo experiments including echocardiography, hemodynamic measurements, and necropsy, were performed by investigators blinded to genetic background of the mice and experimental treatment. Some western blot data was obtained by investigators who know genetic background and experimental treatment in order to know in which order the samples needed to be loaded on the gels. All other in vitro experiments were performed in a non-blinded manner because the experiments needed multiple treatments with clear labels for the operation.

Reporting for specific materials, systems and methods

We require information from authors about some types of materials, experimental systems and methods used in many studies. Here, indicate whether each material, system or method listed is relevant to your study. If you are not sure if a list item applies to your research, read the appropriate section before selecting a response.

Materials & experimental systems

n/a	Involved in the study
☐	☒ Antibodies
☐	☒ Eukaryotic cell lines
☒	☐ Palaeontology and archaeology
☐	☒ Animals and other organisms
☒	☐ Clinical data
☒	☐ Dual use research of concern

Methods

n/a	Involved in the study
☒	☐ ChIP-seq
☒	☐ Flow cytometry
☒	☐ MRI-based neuroimaging

Antibodies

Antibodies used

The following commercial antibodies were used at the indicated dilutions:
Rabbit monoclonal Bcl-xL antibody (#2764) (1:6,000) (Cell Signaling Technology)
Rabbit cleaved caspase-3 antibody (#9661) (1:2,000) (Cell Signaling Technology)
Rabbit cleaved caspase-9 antibody (#9507) (1:2,000) (Cell Signaling Technology)
Rabbit monoclonal p44/42 MAPK (Erk1/2) antibody (#9102) (1:5,000) (Cell Signaling Technology)
Rabbit monoclonal phospho-p44/42 MAPK (Erk1/2) (Thr202/Tyr204) antibody (#4370) (1:5,000) (Cell Signaling Technology)
Rabbit polyclonal phospho-GSK-3alpha/beta (Ser21/9) antibody (#9331) (1:3,000) (Cell Signaling Technology)
Rabbit monoclonal GSK-3alpha/beta antibody (#5676) (1:5,000) (Cell Signaling Technology)
Rabbit polyclonal phospho-Akt (Ser473) antibody (#9271) (1:4,000) (Cell Signaling Technology)
Rabbit polyclonal Akt antibody (#9272) (1:8,000) (Cell Signaling Technology)
Rabbit monoclonal phospho-MST1 (Thr183)/MST2 (Thr180) antibody (#49332) (1:1,000) (Cell Signaling Technology)
Rabbit monoclonal NFAT3 antibody (#2183) (1:1,000) (Cell Signaling Technology)
Rabbit monoclonal GAPDH antibody (#5174) (1:8,000) (Cell Signaling Technology)
Rabbit monoclonal Histone H3 antibody (#4499) (1:10,000) (Cell Signaling Technology)
Anti-mouse or -rabbit IgG, HRP-linked antibodies (#7076 and #7074) (1:5,000) (Cell Signaling Technology)
Mouse alpha-actinin (sarcomeric) antibody (#A7811) (1:4,000) (Sigma-Aldrich)
Rabbit monoclonal alpha-tubulin antibody (T6199) (1:8,000) (Sigma-Aldrich)
Mouse monoclonal IP3R-II antibody (Santa Cruz Biotechnology #sc-398434) (1:1,000)
Rabbit polyclonal H-Ras antibody (C-20) (Santa Cruz Biotechnology #sc-520)(1:1,000)
Mouse monoclonal MST1 antibody (BD Transduction Laboratories #611052) (1:4,000)
Mouse monoclonal cardiac Troponin T antibody (Invitrogen, Thermo Fisher Scientific, #MA5-12960) (1:100)
For detection of phosphorylation of Bcl-xL at Ser14, a polyclonal phosphorylation-specific antibody was generated by immunizing rabbits with a phospho-peptide as described previously (1:1,000) (reference 3 (Del Re, D.P. et al. Mol Cell 2014) of this paper)

Validation

All antibodies used are commercially available and were previously validated except rabbit polyclonal phospho-Bcl-xL (Ser14) antibody. All commercially available antibodies have been validated by the manufacturers for their specificity by using knockouts/knockdowns, for their reactivity in the species, and for their compatibility to be used with the respective application. Besides this, the antibodies have been further validated in publications citing the antibodies.

Bcl-xL antibody: https://www.cellsignal.com/products/primary-antibodies/bcl-xl-54h6-rabbit-mab/2764
Cleaved caspase-3 antibody: https://www.cellsignal.com/products/primary-antibodies/cleaved-caspase-3-asp175-antibody/9661?bvstate=pg:2/ct:r
Cleaved caspase-9 antibody: https://www.cellsignal.com/products/primary-antibodies/cleaved-caspase-9-asp353-antibody/9507
p44/42 MAPK (Erk1/2) antibody: https://www.cellsignal.com/products/primary-antibodies/p44-42-mapk-erk1-2-antibody/9102
phospho-p44/42 MAPK (Erk1/2) (Thr202/Tyr204) antibody: https://www.cellsignal.com/products/primary-antibodies/phospho-p44-42-mapk-erk1-2-thr202-tyr204-d13-14-4e-xp-rabbit-mab/4370
GSK-3alpha/beta antibody: https://www.cellsignal.com/products/primary-antibodies/gsk-3a-b-d75d3-rabbit-mab/5676
phospho-GSK-3alpha/beta (Ser21/9) antibody: https://www.cellsignal.com/products/primary-antibodies/phospho-gsk-3a-b-ser21-9-antibody/9331
phospho-Akt (Ser473) antibody: https://www.cellsignal.com/products/primary-antibodies/phospho-akt-ser473-antibody/9271
Akt antibody: https://www.cellsignal.com/products/primary-antibodies/akt-antibody/9272
phospho-MST1 (Thr183)/MST2 (Thr180) antibody: https://www.cellsignal.com/products/primary-antibodies/phospho-mst1-thr183-mst2-thr180-e7u1d-rabbit-mab/49332
NFAT3 antibody: https://www.cellsignal.com/products/primary-antibodies/nfat3-23e6-rabbit-mab/2183
GAPDH antibody: https://www.cellsignal.com/products/primary-antibodies/gapdh-d16h11-xp-rabbit-mab/5174
Histone H3 antibody: https://www.cellsignal.com/products/primary-antibodies/histone-h3-d1h2-xp-rabbit-mab/4499
Anti-mouse IgG, HRP-linked antibody: https://www.cellsignal.com/products/secondary-antibodies/anti-mouse-igg-hrp-linked-antibody/7076
Anti-rabbit IgG, HRP-linked antibodies: https://www.cellsignal.com/products/secondary-antibodies/anti-rabbit-igg-hrp-linked-antibody/7074
alpha-actinin (sarcomeric) antibody: https://www.sigmaaldrich.com/US/en/product/sigma/a7811?gclid=Cj0KCQjwz8emBhDrARIsANNJjS6fnYglJDkgfXk21qCLlHuzPgqvpvhCNV4MBfjd05jJBIl_1yq261saApksEALw_wcB&gclsrc=aw.ds
alpha-tubulin antibody: https://www.sigmaaldrich.com/US/en/product/sigma/t6199
IP3R-II antibody: https://www.scbt.com/p/ip3r-ii-antibody-a-5?gclid=Cj0KCQjwz8emBhDrARIsANNJjS52kodM0HZosBkM3Anv4tXBE35eHpaPfnGPZvQK94UIeGpgc8W5czQaAqh1EALw_wcB
H-Ras antibody (C-20): https://www.scbt.com/p/h-ras-antibody-c-20?requestFrom=search

MST1 antibody: https://www.bdbiosciences.com/en-us/products/reagents/microscopy-imaging-reagents/immunofluorescence-reagents/purified-mouse-anti-human-mst1.611052
Cardiac Troponin T antibody: https://www.thermofisher.com/antibody/product/Cardiac-Troponin-T-Antibody-clone-13-11-Monoclonal/MA5-12960

Custom phosphorylation-specific antibody against Bcl-xL at Serine 14 was validated for specificity by using a phosphorylation-resistant mutant or appropriate experimental controls whenever possible (PMID: 24813943, PMID: 27218122).

Eukaryotic cell lines

Policy information about cell lines and Sex and Gender in Research

Cell line source(s)	HEK293 cells obtained from the American Type Culture Collection (CRL-1573).
Authentication	Cell line used have been authenticated by the provider by using cell morphology and Karyotyping.
Mycoplasma contamination	The cell line tested negative for Mycoplasma by the provider.
Commonly misidentified lines (See ICLAC register)	No commonly misidentified lines were used in this study.

Animals and other research organisms

Policy information about studies involving animals; ARRIVE guidelines recommended for reporting animal research, and Sex and Gender in Research

Laboratory animals	All mice included in this study were on a C57Bl/6J genetic background. Mouse strains used in this study include Bcl-xL Ser14Ala knock-in mice (reference 4 (Nakamura, M. et al. JCI Insight 2016) of this paper) and C57Bl/6J wild-type mice purchased from Jackson Labs. All mice used in this study ranged from 2 to 4 months of age depending on the experiments. One-day-old Crl:(WI)BR-Wistar rats (Envigo, Somerville) were used for isolation of primary cultures of neonatal ventricular cardiomyocytes. Mice were housed under a specific pathogen-free condition in up to 5 mice per cage, and maintained in a temperature and humidity-controlled environment within a range of 21 °C - 23 °C and 30 - 70%, respectively, with a 12-hour light/dark cycles, in which they received food and water ad libitum. Handling of mice and euthanasia with CO_2 in an appropriate chamber were conducted in accordance with guidelines on euthanasia of the American Veterinary Medical Association. Rutgers is accredited by AAALAC International, in compliance with Animal Welfare Act regulations and Public Health Service (PHS) Policy on Humane Care and Use of Laboratory Animals, and has a PHS Approved Animal Welfare Assurance with the NIH Office of Laboratory Animal Welfare (D16-00098 (A3158-01)).
Wild animals	No wild animals were used in this study.
Reporting on sex	In general, the estrogen and its cycle in female mice impact on studying hypertrophy and heart failure. In addition, the mortality and morbidity of cardiovascular diseases are sex-dependent. Therefore, only male mice were used in our animal studies. Both sexes of rats were used to isolate neonatal ventricular cardiomyocytes since it is difficult to separate male and female neonates.
Field-collected samples	No field-collected samples were used in this study.
Ethics oversight	All experimental procedures with mice were approved by the Institutional Animal Care and Use Committee at Rutgers New Jersey Medical School, under protocol IACUC PROTO999900919 and 201900140.

Note that full information on the approval of the study protocol must also be provided in the manuscript.
